# Research progress on the cannabinoid type-2 receptor and Parkinson’s disease

**DOI:** 10.3389/fnagi.2023.1298166

**Published:** 2024-01-08

**Authors:** Xiaoqi Yu, Yi Jia, Yuan Dong

**Affiliations:** ^1^Neuropsychiatry Research Institute, The Affiliated Hospital of Qingdao University, Qingdao University, Qingdao, China; ^2^School of Basic Medical Sciences, Qingdao University, Qingdao, China

**Keywords:** Parkinson’s disease, CB_2_ receptor, mitochondrial function, neuroinflammation, iron transport

## Abstract

Parkinson’s disease (PD) is featured by movement impairments, including tremors, bradykinesia, muscle stiffness, and imbalance. PD is also associated with many non-motor symptoms, such as cognitive impairments, dementia, and mental disorders. Previous studies identify the associations between PD progression and factors such as α-synuclein aggregation, mitochondrial dysfunction, inflammation, and cell death. The cannabinoid type-2 receptor (CB_2_ receptor) is a transmembrane G-protein-coupled receptor and has been extensively studied as part of the endocannabinoid system. CB_2_ receptor is recently emerged as a promising target for anti-inflammatory treatment for neurodegenerative diseases. It is reported to modulate mitochondrial function, oxidative stress, iron transport, and neuroinflammation that contribute to neuronal cell death. Additionally, CB_2_ receptor possesses the potential to provide feedback on electrophysiological processes, offering new possibilities for PD treatment. This review summarized the mechanisms underlying PD pathogenesis. We also discussed the potential regulatory role played by CB_2_ receptor in PD.

## Introduction

**Figure 1:**
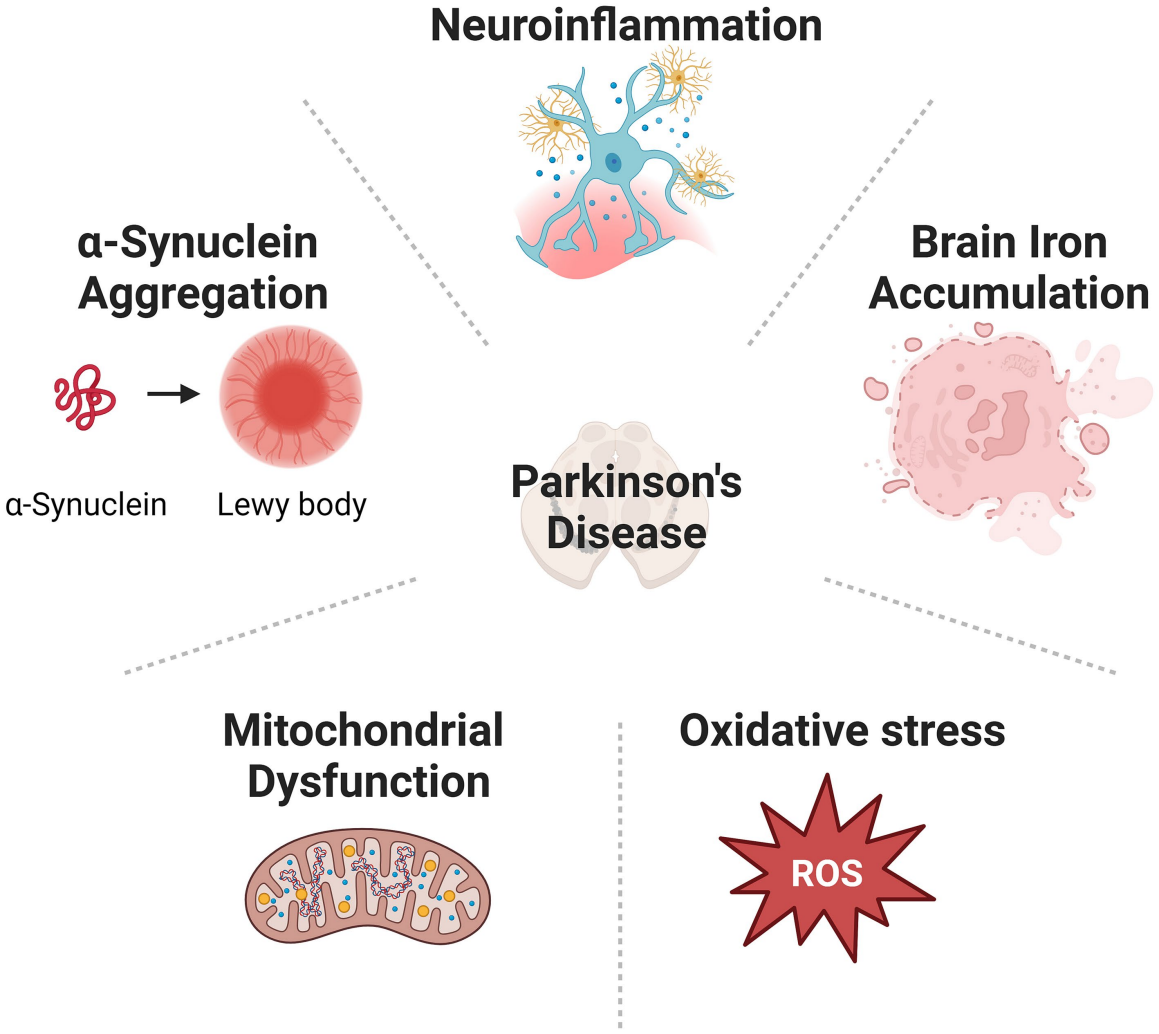
Cause of PD is various, including α-synuclein aggregation, mitochondrial dysfunction, oxidative stress, abnormal iron accumulation, and neuroinflammation.

Parkinson’s disease (PD) is one of the most prevalent neurodegenerative diseases (de Lau and Breteler, 2006; Subramaniam and Chesselet, 2013). Patients with PD are commonly suffering from movement disorders, such as tremors, involuntary movements, rigidities, and imbalance. Many patients also demonstrate non-movement disorders, including cognitive impairments, sleep disorder, chronic pain, olfactory dysfunction, anxiety, and depressive disorder (Garcia-Ruiz et al., 2014; Tolosa et al., 2021). Many patients diagnosed with PD eventually develop dementia during the advanced stage (Szeto et al., 2020). The main pathological feature of PD includes gradual loss of dopaminergic (DA) neurons in the substantia nigra pars compacta (SNpc) located at the midbrain, and the accumulation of Lewy bodies (LBs) containing mainly α-synuclein (α-syn) intracellular inclusions all over the brain (Warren et al., 2017). Multiple mechanisms, including α-syn aggregation (Roy, 2017), mitochondrial dysfunction (Subramaniam and Chesselet, 2013), oxidative stress, abnormal iron accumulation (Hare and Double, 2016), and neuroinflammation (Gelders et al., 2018), have been implicated in the neurodegenerative process of PD (Figure 1). However, the exact cause of PD is still not clear. Consequently, clinical therapies for PD treatment, including medicines and surgeries, are mostly symptomatic. No treatment can stop or reverse the development of PD. The endocannabinoid system (ECS) comprises a network of endocannabinoids (eCBs) and their receptors that are widespread throughout the central nervous system (CNS) and immune system. This tightly regulated system modulates the transmission of chemical signals via an immediate feedback mechanism. Dysregulation of eCB signalling has been suggested in the development of neuropsychiatric disorders and neurodegenerative disease (Yin et al., 2019; Cooray et al., 2020). eCBs are recognized by cannabinoid receptors (CB receptors): the cannabinoid type-1 receptor (CB_2_ receptor) and the cannabinoid type-2 receptor (CB_2_ receptor). Among them, the CB_2_ receptor is mainly located in immune cells. Its activation is reported to exert protective effects in neurological disorders and thus receives extensive attention as a new treatment target. Here, we summarized the current research progress of how the CB_2_ receptor is involved in the pathogenesis and progression of PD and discussed the potential of targeting the CB_2_ receptor for the treatment of this disease.

### The endocannabinoid system (ECS)

Cannabinoids, as an emerging therapeutic agent, have attracted wide attention for their great potential in the treatment of various diseases. They are best understood for their inhibitory effects on the release of γ-aminobutyric acid (GABA) and glutamate through CB_1_ and CB_2_ receptors (Urits et al., 2020). The ECS consists of two major branches: the CB_1_ receptor is highly enriched in the brain and its surrounding nerves (Herkenham et al., 1991), meanwhile, the CB2 receptor is mainly found in the immune system (Facci et al., 1995). Cannabinoids are generally classified into three types based on their source: phytocannabinoids (found in cannabis plants, for example, Δ^9^-tetrahydrocannabinol, THC), synthetic cannabinoids (chemically synthesized), and endocannabinoids (eCBs, i.e., naturally occurring in the human body). Cannabinoids bind to CB receptors located on the cell membrane, exerting corresponding psychotropic effects (Howlett et al., 1990). The eCBs, CB receptors, and enzymes catalyze the synthesis and degradation collectively form the ECS. The activation of the ECS is related to decreased dopaminergic activity and can regulate various neural functions related to emotions, cognitions, motor controls, feeding behaviors, and pain (Castillo et al., 2012; Pacher and Kunos, 2013).

#### The eCBs

N-arachidonoylethanolamine (anandamide, AEA) and 2-arachidonoylglycerol (2-AG), which share highly similar structures with Δ^9^-THC, are the two major and most well-understood eCBs. Generally, they are released from the postsynaptic terminal after neuronal activation, modulate presynaptic neurotransmissions, and produce physiological feedback mechanisms dedicated in preventing excessive excitation of neurons (Lovinger, 2008; Zou and Kumar, 2018). This retrograde feedback initiates depolarization-induced suppression of inhibition (DSI) at GABAergic synapses and depolarization-induced suppression of excitation (DSE) at glutamatergic synapses (Makara et al., 2005). AEA and 2-AG, unlike other neurotransmitters and neuropeptides that are stored in the intracellular compartments, are produced on demand from the cleavage of their precursors, N-arachidonyl-phosphatidyl ethanolamine (NAPE) and diacylglycerol (DAG), respectively (Maccarrone and Finazzi-Agro, 2003).

In the CNS, the eCBs are synthesized by both neuronal cells and glial cells such as microglia (Kelly et al., 2020). *In vitro* study reveals the production of both AEA and 2-AG by microglia (Walter et al., 2003; Carrier et al., 2004). Adenosine triphosphate (ATP) stimulation of microglia increases the production of 2-AG through the activation of P2X purinoceptor 7 (P2X7) ionotropic receptor (Witting et al., 2004). Microglia is suggested as the one of the main source of eCBs under neuroinflammation (Stella, 2009). Upregulated eCB levels are implicated in anti-inflammatory effects, and therefore are believed to exert neuroprotective effects in various diseases. 2-AG is reported to limit acute neuroinflammation induced by the Theiler’s murine encephalomyelitis virus (TMEV) by modulating microglial activation and promoting the activation of brain-derived suppressor cells, indicating a potent regulatory function of 2-AG on peripheral and central immunity (Mecha et al., 2018). AEA treatment is found to attenuate the lipopolysaccharide (LPS)-induce microglia activation via the CB_2_ receptor (Malek et al., 2015). Clinical study recently reveals that deficiency of diacylglycerol lipase β (*DAGLB*), the synthase of 2-AG, is associated with early onset of PD. Knockdown of *Daglb* impairs locomotor skill learning in mice (Liu et al., 2022). In 1-methyl-4-phenyl-1,2,3,6-tetrahydropyridine (MPTP)-induced PD mouse model, increased level of 2-AG is reported in the ventral midbrain after MPTP treatment (Mounsey et al., 2015). Exogenous addition of 2-AG or monoacylglycerol lipase (MAGL, enzyme for 2-AG hydrolysis) inhibitors demonstrate potent protective effect against MPTP-induced cell death (Mounsey et al., 2015). Collectively, those studies suggest the potential neuroprotective effects of eCBs in PD via regulation of microglia and neuroinflammation.

#### The CB receptors

Biological effects of the eCBs and other synthetic cannabinoids (such as WIN55,212–2 and HU210) are mainly mediated by the G-protein-coupled CB receptors: the CB_1_ and CB_2_ receptors (Munro et al., 1993). The activation of CB_1_ receptors involves the coupling of pertussis toxin (PTX)-sensitive G proteins (Gαi/o), leading to the inhibition of adenylate cyclase (AC) and cyclic adenosine monophosphate (cAMP) formation. Activation of CB_1_ receptor also activates the mitogen-activated protein kinase (MAPK) and the phosphatidylinositol 3-kinase (PI3K)/protein kinase B (AKT) signaling pathways, which both participate in the regulation of cell proliferation, cell cycle, and cell death (Pertwee, 2006; Howlett et al., 2010; Turu and Hunyady, 2010; Blázquez et al., 2015). CB_1_ receptors can also exert their effects through G protein-dependent or other ligand-dependent mechanisms (Demuth and Molleman, 2006). In addition to the typical G protein-dependent signaling, CB_1_ receptors also transmit signals through interaction with other molecules (such as β-arrestin) in a G protein-independent manner (Howlett et al., 2010). Moreover, CB_1_ receptors also regulate several types of ion channels (Turu and Hunyady, 2010). Upon activation of CB_1_ receptors, the inhibition of Gα_i/o_-mediated cAMP reduction regulates inwardly rectifying potassium channels (GIRKs) and inhibits N-type and P/Q-type voltage-gated calcium channels (Howlett et al., 2002; Fisyunov et al., 2006), thereby suppressing presynaptic neurotransmitter release. Research has shown that CB_2_ receptors regulate the activity of N-type Ca^2+^ channels located at the presynaptic membrane, thereby modulating calcium influx to inhibit GABA release in mouse hippocampal slices (Szabo et al., 2014).

Like the CB_1_ receptor, the CB_2_ receptor is coupled to Gα_i/o_ proteins. However, unlike the CB_1_ receptor, the CB_2_ receptor does not appear to be coupled to potassium channels (McAllister et al., 1999). The CB_2_ receptor is initially thought to be predominantly expressed in the peripheral immune system. However, recent studies have found CB_2_ receptor expression in the CNS (Mackie, 2008). The CB_2_ receptor is expressed by microglia, astrocytes and certain subpopulations of neurons (Fernández-Ruiz et al., 2008). Upregulation of CB_2_ receptor has been implicated in neurodegenerative diseases. Activation of this receptor in animal models demonstrate disease-modifying effects against the process of neurodegeneration, suggesting CB_2_ receptor is a promising therapeutic target for the treatments of such disease. Also, compared to CB_1_ receptor, the activation of CB_2_ receptor has been shown to have fewer psychoactive and other side effects (Pacher et al., 2006; Liu et al., 2021), making selective CB_2_ receptor targeting a better option for this approach. In the following parts, we summarized the current understanding of how CB_2_ receptor participates in the progression of PD, and its potential as a treatment target in the treatment of this disease.

### CB_2_ receptor in PD

#### The role of CB_2_ receptor in PD

Both clinical and animal studies reveal the alternation of CB_2_ receptor in PD. Postmortem studies reveal the increased level of CB_2_ receptor in microglial cells at substantia nigra (SN) of PD patients, indicating the recruitment and activation of microglia at the site of lesion (Gómez-Gálvez et al., 2016). This finding is supported by the observation in animal models of PD. CB_2_ receptor level is significantly increased in both LPS- and 6-hydroxydopamine (6-OHDA)-induced PD model, and this elevation is associated with the activation of microglia (Concannon et al., 2015). Those findings suggest the upregulation of CB_2_ receptor in microglia. However, downregulation of CB_2_ receptor is also reported in neurons and other brain regions. Reduced level of CB_2_ receptor is reported in the tyrosine hydroxylase (TH)-containing in the SN of PD patients, indicating increased DA neuronal cell death (García et al., 2015). Reduced transcription of CB_2_ receptor is observed in the cerebellum and hippocampus of PD patients, as compared to healthy controls (Grünblatt et al., 2007). Similarly, in the MPTP-induced PD mouse model, a downregulation of CB_2_ receptor is observed 3 weeks after MPTP injection (Shi et al., 2017; Xin et al., 2020). Further research demonstrates neuroprotective potentials of CB_2_ receptor in PD. Specifically, CB_2_ receptor-deficient mice demonstrate more severe loss of tyrosine TH-containing neurons in the SN, indicating the protective role of CB_2_ receptor in PD (Gómez-Gálvez et al., 2016). In an *in vitro* PD model established by MPP^+^ treatment, JWH133 (a potent CB_2_ receptor agonist) is shown to promote cell survival (Aymerich et al., 2016). *In vivo* study also demonstrates that the administration of nonselective CB receptor agonist WIN55,212–2 and selective CB_2_ receptor agonist JWH015 alleviate the MPTP-induced neuron death and microglial activation in SN (Price et al., 2009). GW842166x (a selective CB_2_ receptor agonist) exerts protective effects against the 6-OHDA-induced loss of dopamine neurons (Yu et al., 2021). Another selective CB_2_ receptor agonist AM1241 is reported to alleviate the MPTP-induced PD-like symptoms and promote the regeneration of DA neurons in mice (Shi et al., 2017). Moreover, administration of β-caryophyllene (BCP, a CB_2_ receptor agonist) is reported to exert neuroprotective effects in both rotenone (ROT)-induced and MPTP-induced PD animal models (Javed et al., 2016; Viveros-Paredes et al., 2017). Those research findings collectively suggest the potential protective effects of CB_2_ receptor agonist in PD, rising the discussion of targeting CB_2_ receptor as a potential treatment approach for PD. Therefore, we further discuss the potential roles of CB_2_ receptor in PD from different perspectives and possible mechanisms in the following sections.

#### Role of CB_2_ receptor in α-syn pathology

α-syn is one of the major components involved in the formation of LBs. α-syn oligomers exert strong cytotoxic effects to neuron (Ghosh et al., 2017; Calabresi et al., 2023). The formation of α-syn oligomers is influenced by multiple factors. Clinical studies have shown significantly elevated level of α-syn oligomers in the plasma, serum, and red blood cells of PD patients, as compared to healthy controls (Zhao et al., 2022). Interestingly, it has been reported that the peripheral autonomic nervous system may be a key pathway for the spread of α-syn pathology from the periphery to the CNS (Chen et al., 2020). Numerous research and clinical findings reemphasize the central role of α-syn, and α-syn-induced neurotoxicity and neuroinflammation in PD (Fayyad et al., 2019; Wang et al., 2019). However, the interaction between CB_2_ receptor and α-syn has been largely over-looked. Recently, Feng et al. demonstrate that the fibrillar α-syn treatment causes significantly promoted neuroinflammation and phagocytosis, as revealed by higher level of cluster of differentiation 68 (CD68) and interleukin-1β (IL-1β), reduced level of brain-derived neurotrophic factor (BDNF) in mice with CB_2_ receptor knockout, as compared to wild-type (WT) mice (Feng et al., 2023). Indeed, they also find that CB_2_ receptor knockout promotes the activation of microglia and pruning of cholinergic synapses induced by α-syn treatment (Feng et al., 2023), suggesting the important role played by CB_2_ receptor in α-syn pathology.

#### The inhibitory effect of CB_2_ receptor in neuroinflammation

Extensive post-mortem examinations, brain imaging studies, epidemiological data, and animal studies have demonstrated the contribution of innate and adaptive immunities in neurodegeneration (McGeer et al., 1988; Gerhard et al., 2006; Theodore et al., 2008). It is widely believed that the degeneration and death of neurons in neurodegenerative diseases are primarily influenced by the release of inflammatory factors and neurotoxic mediators, such as IL-1β, tumor necrosis factor α (TNF-α), interleukin-6 (IL-6), interleukin-8 (IL-8), interleukin-33 (IL-33), chemokine ligand 2 (CCL2), chemokine ligand 5 (CCL5), prostaglandin E2 (PGE2), cyclooxygenase-2 (COX-2), and increased ROS (Skaper et al., 2014; Kempuraj et al., 2015). These mediators bind to corresponding receptors on neurons or glia cells, directly or indirectly induce neurodegeneration and affect neuronal survival through interactions with neuroglial cells. Meanwhile, the activation of glial cells, including microglial cells and astrocytes, promote the expression of pro-inflammatory mediators in neurodegenerative diseases (Kim and Lee, 2014), causing aggravated neurodegeneration, which further exacerbates the progression of the disease course. In PD, the contribution of neuroinflammation has been intensively studied and suggested as a promising target for effective treatment (Tansey et al., 2022).

CB_2_ receptor has been identified as a potential anti-inflammatory component in various inflammation-related diseases. Its activation disrupts the self-sustained neuroinflammation status that contributes to the disease progression of neurodegeneration. Activation of CB_2_ receptor reduces the release of pro-inflammatory cytokine and thereby prevents neuronal cell death in neurodegeneration diseases. LPS injection in mice leads to an increase in TNF-α levels and oxidative stress in the brain, resulting in disease-like behavior. Acute injection of the CB_2_ receptor agonist 1-phenylisatin (PI) significantly rescues the behavioral changes induced by LPS administration in mice (Sahu et al., 2019). Moreover, PI inhibits the transcription of TNF-α and oxidative stress in the brain, demonstrating that both acute and long-term activation of CB_2_ receptor may exert protective effect against the development of various disease related to neuroinflammation and oxidative stress (Sahu et al., 2019). Activation of CB_2_ receptor is reported to inhibit the activation of NLR Family Pyrin Domain Containing 3 (NLRP3) inflammasome, a potent contributor of neuroinflammation and neurodegenerative diseases (Ke et al., 2016; Yu et al., 2019). In human microglial cells derived from the temporal lobe, JWH015 exerts neuroprotective effects by reducing the release of TNF-α and IL-1β (Klegeris et al., 2003).

Non-selective CB_2_ receptor agonist WIN55,212–2 and selective CB_2_ receptor agonist JWH015 have been shown to reduce MPTP-induced microglial infiltration. This effect can be reversed by the CB_2_ receptor antagonist JTE907, confirming the CB_2_ receptor-mediated inhibitory effect via the modulation of microglia (Price et al., 2009). CB_2_ receptor activation by JWH133 is reported to reduce the level of pro-inflammatory cytokines and promote the M2 polarization of microglia via the activation of the PI3K/Akt signalling pathway (Wang et al., 2023). In the MPTP-induced PD mouse model, CB_2_ receptor knockout exhibits aggrieved microglial activation, along with neuropathology and functional deficits (Komorowska-Muller and Schmole, 2020). In an environmental and viral inflammation-induced PD model established by unilateral intrastriatal injection of ROT or polyinosinic:polycytidylic acid (Poly I:C) in male rats, a significant increase of CB_2_ receptor expression is observed, which is strongly correlated with activated microglia in the model (Concannon et al., 2016). Similarly, in ROT-induced and MPTP-induced PD animal models, CB_2_ receptor agonizing using BCP demonstrates disease-alleviating effect via the suppression of neuroinflammation (Javed et al., 2016). ROT injection leads to microglial activation and subsequent inflammation. Further research has showed that the activation of CB_2_ receptor by BCP can inhibit ROT-induced microglial activation, improve the release and expression of inflammatory mediators in CNS, and attenuate the expression of inflammatory factors such as NF-κB, COX-2, and Inducible nitric oxide synthase (iNOS) (Javed et al., 2016).

Excessive inflammation not only involves the activation of microglial cells but also the activation and proliferation of astrocytes, which play a crucial regulatory role in the inflammatory response. Currently, there is limited research on the effects of CB_2_ receptor on astrocytes. It has been demonstrated that rat astrocytes express both CB_1_ receptor and CB_2_ receptor (Stella, 2004; Sheng et al., 2005). Recent studies also report the colocalization of CB_2_ receptor with astrocytes by immunohistochemical localization. Increased immunoreactivity of CB_2_ receptor in astrocytes is reported in PD patients (Navarrete et al., 2018). This suggests that the expression changes of CB_2_ receptor in astrocytes have potential regulatory roles in PD, and warrant further investigation. In primary cultured astrocytes, the nonspecific CB receptor agonist WIN 55,212–2 has been shown to regulate cell viability, inflammatory mediators, and oxidative stress. Specifically, the amyloid-β (Aβ) 1–42, the aberrant protein aggregation contributes to the pathogenesis of Alzheimer’s disease (AD), reduces astrocyte viability while increasing the expression of TNF-α, IL-1β, COX-2, and iNOS. Meanwhile, pre-treatment with WIN 55,212–2 significantly rescues the inflammatory and astrocyte vulnerability to Aβ1-42 treatment (Aguirre-Rueda et al., 2015). Furthermore, JWH133 is reported to exert neuroprotective effects by inhibiting blood–brain barrier (BBB) damage, astrocytic targeting myeloperoxidase (MPO) expression, peripheral immune cell infiltration, and the production of inflammatory and chemotactic factors by activated microglial cells (Chung et al., 2016). Collectively, those results indicate CB_2_ receptor as a promising disease-modifying treatment target for PD via its regulation of neuroinflammation.

#### The inhibitory effect of CB_2_ receptor on oxidative stress

The motor dysfunction in PD is caused by the loss of DA neurons in the SNpc. Increasing evidence suggests that oxidative stress is a key driving factor in the complex degenerative cascade of dopaminergic neurodegeneration in all forms of PD (Dias et al., 2013; Blesa et al., 2015). Markers of oxidative stress in the CNS increase with aging and the occurrence of neurodegenerative diseases (Boveris and Navarro, 2008). Oxidative stress arises from a disruption in cellular redox homeostasis, where the production of reactive oxygen species (ROS) exceeds the clearance rate by endogenous antioxidant enzymes and molecular chaperones. Uncontrolled oxidative reactions within cells cause destructive damage to normal cellular structures, leading to cellular degeneration and death (Wiseman and Halliwell, 1996; Rego and Oliveira, 2003). Accumulation of ROS induces oxidative damage to lipids, proteins, DNA, and RNA, impairing neuronal function and structural integrity (Schieber and Chandel, 2014). Due to the increased chances of spontaneous mutations resulting from oxidative stress, it may trigger mutations that make cells more susceptible to functional impairments, and the vulnerability of the SN to oxidative stress contributes to selective neuronal degeneration (Floor and Wetzel, 1998). The damaging effects of oxidative stress are well recognized, and research focusing on inhibiting neuronal oxidative stress has become a mainstream direction in PD treatment.

Previous research demonstrates that activation of CB_2_ receptor can protect DA neurons against degeneration in a ROT-induced PD model (Javed et al., 2016). ROT injection causes extensive loss of DA neurons in the SNpc and striatal fibers, leading to oxidative damage characterized by reduction of anti-oxidant enzymes and upregulated nitrite level (Thakur and Nehru, 2013). Treatment with the CB_2_ receptor agonist BCP prevents glutathione depletion, enhances antioxidant enzyme activity in the midbrain, and inhibits the elevation of nitrite levels. It has been found that the GW405833 (a CB_2_ receptor-specific agonist) administration inhibits inflammatory response by suppressing the levels of cytokine and oxidative stress (Parlar et al., 2018). Other research results report that the CB_2_ receptor agonist HU308 reduces the production of ROS-generating enzymes NOX4, NOX2, and NOX1, as well as subsequent renal oxidative stress in mice (Zhang et al., 2009). An *in vitro* study demonstrates that CB_2_ receptor is involved in the antioxidant stress process in RAW264.7 macrophages, blocking cell death (Giacoppo et al., 2017). These results indicate that activation of CB_2_ receptor can inhibit oxidative stress and protect neuronal cells.

#### CB_2_ receptor and iron transport

Excessive accumulation of iron in the brain is a major characteristic of brain degeneration in patients with PD, known as brain iron accumulation. Non-physiological accumulation of iron in specific brain regions is associated with various diseases. This phenomenon is referred to as neurodegeneration with brain iron accumulation (NBIA) (Schneider et al., 2012). It has been reported that iron levels in the SN of PD patients increase significantly. This change is accompanied by upregulation of divalent metal transporter 1 (DMT1), a protein involved in iron transport (Jia et al., 2015). Iron accumulation may exert its pathogenic activity by increasing ROS and causing widespread damage to intracellular proteins. However, there is also evidence suggesting that it leads to neuronal death through interactions with pathological protein aggregates found in these diseases by promoting the process of cellular apoptosis (Ward et al., 2014).

Maintaining iron homeostasis in the brain has long been considered a potential target for drug treatment related to aging-related diseases. Iron is involved in various cellular functions, such as the synthesis of myelin phospholipid, mitochondrial respiration, and the biosynthesis and metabolism of neurotransmitters. Therefore, the regulation of iron transport through DMT1 plays a significant role in maintaining normal brain physiological function. It has been reported that Δ^9^-THC, CP 55940, WIN 55,212–2, and AEA inhibit the uptake of 55Fe and 54Mn in HEK293T cells expressing DMT1 by stabilizing the expression of the transporter protein and inhibiting DMT1 expression. Small-molecule tests have shown that Δ9-THC inhibits DMT1 activity (Wetli et al., 2006). Furthermore, gene knockout of the CB_2_ receptor eliminates its regulatory effects, indicating that the inhibitory effect of Δ9-THC is mediated by the CB_2_ receptor. Moreover, activation of CB_2_ receptor negatively regulates signaling cascades related to serine/threonine kinases. Immunoprecipitation experiments have shown that phosphorylation of serine 43 of DMT1 promotes its transport activity, thereby facilitating iron absorption. Δ^9^-THC blocks serine phosphorylation of DMT1, and CB_2_ receptor knockout abolishes the blockade of iron transport by Δ^9^-THC (Seo et al., 2016).

#### The regulatory effect of CB_2_ receptor on mitochondrial function

Mitochondria play a pivotal role in the vitality of eukaryotic cells as they are involved in bioenergetics, metabolism, and signaling, and are associated with many diseases (Pfanner et al., 2021). The involvement of mitochondrial dysfunction in the pathogenesis of PD is discovered when individuals who consumed illegally contaminated drugs containing MPTP developed PD-like symptoms (Langston et al., 1983). It has been demonstrated that mitochondrial dysfunction can induce degeneration and death of DA neurons (More and Choi, 2015), promoting the occurrence of neurodegenerative in PD (Bose and Beal, 2016).

Previous research has shown that cannabinoids such as Δ^9^-THC and synthetic cannabinoid HU210 impair mitochondrial respiratory function via the suppression of oxygen consumption and mitochondrial membrane potential (ΔΨm) (Athanasiou et al., 2007). ΔΨm manifests the functional status of mitochondria. Additionally, both AEA and 2-AG suppress the transcription of genes associated with mitochondrial biogenesis, and decrease mitochondrial DNA content and oxygen consumption in white adipocytes of mouse (Tedesco et al., 2010). Further studies have found that activation of CB_2_ receptor using JWH133 conveys an anti-apoptotic effect in animal model of myocardial ischemia (Li et al., 2013), which aligns with the protective outcome of JWH133 against ischemia-induced ΔΨm loss and cytochrome c release from mitochondria to the cytoplasm. Moreover, CB_2_ receptor is involved in AEA-stimulated mitochondrial cation transport (Zoratti et al., 2003). Collectively, CB_2_ receptor is believed to play a regulatory role in modulating mitochondrial respiratory activity. How this regulatory effect of CB_2_ receptor related to PD is therefore worth further investigation.

#### CB_2_ receptor and autophagy

Autophagy is a lysosome-dependent self-degradation and recycling process. It is an essential metabolic process that targets protein and dysfunctional cellular components (Kim and Lee, 2014; Saha et al., 2018). Autophagy is a conserved cellular process that maintains cellular homeostasis. Autophagy impairments are closely related to the pathogenesis of PD (Cheng et al., 2020; Lu et al., 2020). Further studies reveal the association between autophagy and CB_2_ receptor. It has been demonstrated that autophagy is related to the protective functions of CB_2_ receptor in several diseases (Shao et al., 2014; Denaës et al., 2016). Ke et al. (2016) found that activation of CB_2_ receptor alleviates the effects of NLRP3 inflammasome activation by inducing autophagy in rat macrophages, thereby reducing inflammation in a mouse model of inflammatory bowel disease (IBD). Additionally, there is a similar association between CB_2_ receptor and autophagy in a mouse model of multiple sclerosis. It has been shown in mice that activation of CB_2_ receptor can induce autophagy to prevent diabetic cardiomyopathy (Wu et al., 2018). These studies suggest that inducing autophagy through the activation of CB_2_ receptor has potential therapeutic value in the progression of PD.

#### The electrophysiological regulatory effects of CB_2_ receptor

There is electrophysiological evidence suggesting that activation of CB_2_ receptors can regulate neuronal activity and excitability. CB_2_ receptors have been found to be expressed in ventral tegmental area (VTA) DA neurons (Foster et al., 2016), and systemic and local administration of JWH133 has been shown to enhance M-type potassium currents, leading to neuronal inhibition and hyperpolarization, significantly reducing the firing frequency of VTA DA neurons both *in vivo* and *in vitro* (Zhang et al., 2014, 2017). Specifically, in whole-cell perforated and cell-attached membrane patch clamp recordings from individual neurons or brain slices in wild-type mice, JWH133 dose-dependently suppressed the firing of VTA DA neurons, and this effect is blocked by AM630 and observed in CB_2_ receptor knockout mice. Similar effects have also been observed in rats, indicating that activation of CB_2_ receptor in the brain can regulate the firing of VTA DA neurons, exerting electrophysiological regulatory effects and providing new avenues for the treatment of PD.

#### The neuroprotective effects of CB_2_ receptor on DA neuron

Given that the main characteristic of PD is the loss of DA neurons in SN and a significant reduction in striatal dopamine, the current mainstay of PD clinical treatment involves the use of levodopa (L-DOPA). However, long-term use of L-DOPA often leads to fluctuations and motor complications that offset its beneficial effects (Utsumi et al., 2013). Therefore, many studies are focused on developing novel non-dopaminergic drugs that can prevent or even reverse the degeneration of DA neurons. CB_2_ receptors are detected in central nervous system regions including the striatum, hippocampus, basal ganglia, frontal cortex, amygdala as well as the VTA (Morris et al., 2021), and their activation is involved in various diseases associated with DA neuron injuries. Mice overexpressing CB_2_ receptor show significantly reduced damage to DA neurons induced by 6-OHDA, reduced motor impairment, and decreased activation of glial cells in the affected area (Ternianov et al., 2012). Activation of CB_2_ receptor using the CB_2_ receptor agonist AM1241 can protect against MPTP-induced PD mouse models, leading to an increase in the number of TH-positive cells in the SN, indicating the regeneration of DA neurons in PD mice and suggesting AM1241 as a potential candidate for PD treatment (Shi et al., 2017). Research data obtained from DA neuron-specific CB_2_ receptor knockout mice indicates that the absence of CB_2_ receptor in DA neurons modulate psychomotor and reward behavior (Liu et al., 2017). This further confirms the protective functions of CB_2_ receptor on DA neurons and establishes a new target for PD treatment.

#### CB_2_ receptor prevents motor dysfunction

Motor dysfunction is a prominent feature in the progression of PD and poses significant inconvenience and harm to patients (Bologna et al., 2020). In PD models established by unilateral lesion of DA neurons, induced by 6-OHDA or LPS injection in male Sprague Dawley rats, behavioral tests for motor dysfunction and CB_2_ receptor detection are conducted on days 7, 14, and 28. The animal exhibits motor dysfunction, and the expression of CB_2_ receptor is significantly upregulated in the PD models (Concannon et al., 2015). Previous studies have found that activation of CB_2_ receptor using agonists improve certain aspects of motor dysfunction, providing a solution to alleviate the motor deficits caused by PD. In C57BL mice, treatment with the CB_2_ receptor agonist JWH015 alleviates anxiety-like behavior during chronic mild stress, while AM630 enhances anxiety-like behavior (Ishiguro et al., 2018). An increase in CB_1_ and CB_2_ receptor expression in the striatum has been reported in chronic L-DOPA treatment for motor dysfunction, and a correlation between motor dysfunction, striatal activation, and microglial cell activation in the PD model after L-DOPA treatment (Navarro et al., 2018). In a mouse model of PD induced by MPTP treatment, treatment with AM1241 can mitigate weight loss, attenuate MPTP-induced motor impairment, and reduce climbing time in mice (Shi et al., 2017). This indicates the critical role of CB_2_ receptor in preventing MPTP toxicity and highlights the significant therapeutic value of the CB_2_ receptor agonist AM1241 in PD, including the potential regeneration of dopaminergic neurons following neurotoxicity induced by MPTP.

### Therapeutic potential of CB_2_ receptor agonists

Currently, no selective CB_2_ receptor drug has been approved for the treatment of PD. However, several studies have proposed the use of cannabinoids in the treatment of PD (Stampanoni Bassi et al., 2017; Buhmann et al., 2019). In preclinical studies, different phytocannabinoids has demonstrated potent neuroprotective effect in animal models of PD and other neurodegenerative diseases. Phytocannabinoid Δ^9^-tetrahydrocannabivarin (Δ^9^-THCV), a potent agonist of CB_2_ receptor and antagonist of CB_1_ receptor, is reported to attenuate the loss of TH-containing neurons in the SN caused by 6-OHDA administration (García et al., 2011). Similar effect of Δ^9^-THCV is also reported in the PD animal model induce by L-DOPA (Espadas et al., 2020). However, its low BBB-permeability largely limits its application in clinic (Deiana et al., 2012). BCP, a phytocannabinoid and CB_2_ receptors agonist, is demonstrated to attenuates oxidative stress, neuroinflammation and apoptosis, and produces neuroprotective effects in PD animal models (Javed et al., 2016; al-Taee et al., 2019). Moreover, Δ^9^-THC has been shown to reduce agitation in the late stages of AD (Walther et al., 2006). In 2003, the FDA granted a patent for cannabinoids as antioxidants and neuroprotectants, but their clinical application in PD is yet to be determined (Krishnan et al., 2009).

Studies using synthetic cannabinoids recently have brought new exciting news in this research area. Nabilone, a synthetic form of Δ^9^-THC, mimicking the structure and pharmacological activities of Δ^9^-THC via both CB_1_ and CB2 receptors. This drug is approved by the U.S. Food and Drug Administration (FDA) for treatment of nausea and vomiting caused by chemotherapy. Recently, 2 clinical trials using Nabilone for the treatment of the non-motor symptoms of PD patients have completed (NCT03769896; NCT03773796). The obtained results indicate that Nabilone is able to produce beneficial effects on sleep disorders associated with PD (Peball et al., 2019, 2020, 2022).

## Conclusion and perspective

As a progressive neurodegenerative disorder, the prevalence of PD significantly increases in the past decades. Meanwhile, the incidence of PD is also demonstrating a trend of early onset at younger ages. Intensive studies unravel multiple theories that contributes to the pathogenesis of PD. However, the fundamental mechanisms are not fully understood. Consequently, the current treatment for PD is most symptomatic. For this reason, identifying effective therapeutic targets for PD is critically important. The discovery of CB_2_ receptor by Munro in 1993 (Munro et al., 1993) and subsequent evidence of CB_2_ receptor expression in the brain and neurons of rodents and primates (Zhang et al., 2014; Stempel et al., 2016), as well as alterations in CB_2_ receptor expression in PD, have led to investigations in this area. CB_2_ receptor, as an important component of ECS, plays a protective role in various neurodegenerative diseases (Jordan and Xi, 2019). Selective activation of CB_2_ receptor regulates mitochondrial function, inhibits oxidative stress, suppresses the release of inflammatory factors, and involves in various regulations such as iron transport, electrophysiology, and autophagy. CB_2_ receptor agonists have emerged as promising neuroprotective drugs with considerable therapeutic potential (Spinelli et al., 2017). However, many questions about CB_2_ receptor and its function in PD still remain open, which potentially limits the development and application of the CB_2_ receptor-targeting therapy. First, most of the current research on the neuroprotective effects of CB_2_ receptor has focused on its anti-inflammatory properties in microglia and astrocytes. As neurons also express CB_2_ receptor, its function in neuron and association with neurodegeneration warrant further studies. Second, clinical researches intensively focus on the use of phytocannabinoids such as Δ^9^-THC in the treatment of neurodegenerative disease such as AD. As those compounds are potent agonist of both CB_1_ and CB_2_ receptors, further in-depth clinical research of selective CB_2_ receptor agonists is necessary to fully understand the therapeutic potential of CB_2_ receptor in PD. Finally, the function of CB_2_ receptor in PD is generally believed as neuroprotective and anti-inflammatory, and results little or no adverse CNS effects. However, giving its abundance in the immune system, further investigation of the potential adverse effects of CB_2_ receptor agonizing is critically important for the clinical application of selective CB_2_ receptor agonists.

## Author contributions

XY: Writing – original draft. YJ: Writing – original draft. YD: Funding acquisition, Writing – review & editing.
